# Novel mechanisms underlying anti-polycystic ovary like syndrome effects of electroacupuncture in rats: suppressing SREBP1 to mitigate insulin resistance, mitochondrial dysfunction and oxidative stress

**DOI:** 10.1186/s40659-020-00317-z

**Published:** 2020-10-27

**Authors:** Yan Peng, Xinming Yang, Xi Luo, Chunhong Liu, Xia Cao, Hongyan Wang, Liyuan Guo

**Affiliations:** 1 Disease Prevention Center, The First Affiliated Hospital, Heilongjiang University of Chinese Medicine, Harbin, 150040 People’s Republic of China; 2 Department of Obstetrics and Gynecology, The First Affiliated Hospital, Heilongjiang University of Chinese Medicine, Harbin, 150040 People’s Republic of China; 3 College of Basic Medicine Sciences, Heilongjiang University of Chinese Medicine, Harbin, 150040 People’s Republic of China; 4 Document Retrival Center, Heilongjiang University of Chinese Medicine, Harbin, 150040 People’s Republic of China; 5 Department of Gynecological Oncology, Cancer Hospital of Harbin Medical University, Harbin, 150081 People’s Republic of China

**Keywords:** Electroacupuncture, PCOS, SREBP1, AMPK, Insulin resistance

## Abstract

**Background:**

Acupuncture, a therapy of traditional Chinese medicine, is confirmed to exert the therapeutic action on polycystic ovary syndrome (PCOS). However, the detailed therapeutic mechanisms of acupuncture in PCOS remain ambiguous. In this study, we further investigated whether electroacupuncture (EA) alleviated PCOS-like symptoms in rats via regulating a metabolic regulator, sterol regulatory element binding protein-1 (SREBP1).

**Methods:**

The PCOS-like rat model was built by hypodermic injection with dehydroepiandrosterone (DHEA). The rats were subjected to EA intervention (ST29 and SP6 acupuncture points) for 5 weeks. Primary granulosa cells were isolated from control and PCOS-like rats for evaluating insulin resistance, mitochondrial dysfunction and oxidative stress in vitro.

**Results:**

The expression of SREBP1 was increased in PCOS-like rats, which was suppressed by EA treatment. In addition, lentivirus-mediated overexpression of SREBP1 restrained EA treatment-induced improvement in pathological changes, serum hormone levels and insulin resistance in rats. In addition, overexpression of SREBP1 repressed insulin-stimulated phosphorylation of insulin receptor β (IR) and AKT in primary granulosa cells. Moreover, upregulation of SREBP1 further exacerbated mitochondrial dysfunction and oxidative stress in granulosa cells isolated from PCOS-like rats. Mechanically, EA treatment suppressed SREBP1 expression through inducing the activation of AMP-activated protein kinase (AMPK) signaling pathway in PCOS-like rats.

**Conclusion:**

EA intervention alleviated PCOS-like symptoms in rats via improving IR, mitochondrial dysfunction and oxidative stress through regulating SREBP1, a lipid metabolism regulator. Our findings illuminate the novel protective mechanisms of EA in the treatment of PCOS.

## Background

Polycystic ovary syndrome (PCOS) is an endocrine disorder commonly seen in women of reproductive age, which is considered as the main reason for menstrual disorder and infertility [1]. It was reported that the prevalence rate of PCOS was 6–18% around the world [2]. Numerous studies have confirmed that insulin resistance may frequently occur in PCOS patients and lead to hyperinsulinemia, which takes part in the pathogenic mechanisms of PCOS [3, 4]. Insulin is a kind of multifunctional hormone that is involved in the regulation of glucose, fat, and protein metabolism. Therefore, the insulin resistance in PCOS patients may increase the risk of developing type 2 diabetes mellitus, hypertension, and hyperlipemia [5, 6]. Currently, there are limited available treatments for PCOS. Thus, exploring novel effective measure for PCOS and elucidating its mechanisms are urgently needed.

Previous studies indicated that the pharmacological and surgical therapies for PCOS may inevitably have side effects such as cardiovascular disease and multifetation [7, 8]. However, electroacupuncture (EA) has been recognized as a safe treatment and contributes to restore reproductive endocrine function of PCOS [9–11]. But so far, the therapeutic mechanisms of EA in PCOS has not been clearly clarified.

Sterol regulatory element binding protein-1 (SREBP1) is a key gene of lipid metabolism regulation, and its level has been reported to be upregulated in PCOS patients [12]. Additionally, Shah et al. found that eicosapentaenoic acid alleviated PCOS via suppressing SREBP1 expression in rats [13]. It has also been suggested that SREBP1 plays crucial roles in modulating insulin resistance. For example, inhibition of SREBP1-mediated lipogenesis effectively improved diet-induced insulin resistance [14]. In addition, SREBP1 has been demonstrated to participate in palmitate acid-induced insulin resistance via regulating IRS-1 expression and insulin signaling pathway in skeletal muscle [15]. A previous study by Li et al. showed that EA intervention could attenuate the lipid metabolism disturbance in insulin resistance rats via decreasing SREBP1 expression [16]. However, whether EA can improve PCOS via regulating SREBP1 expression has not been determined, which needs to be expounded.

In this study, for the first time, we evaluated the effect of EA on the abnormally elevated SREBP1 level in a rat model of PCOS-like symptoms and its role in improving insulin resistance, mitochondrial dysfunction and oxidative stress.

## Material and methods

### Animal model

Female Sprague Dawley rats at 4 weeks old were purchased from Chang Sheng biotechnology co., Ltd. (Liaoning, China). The rats were maintained at 25 ± 1 °C, humidity of 45–55%, under12 h light/ 12 h dark cycles and took food and water freely. There were three experimental groups (n = 6 per group): control, PCOS, PCOS + EA. The rats in PCOS group were received daily hypodermic injection with dehydroepiandrosterone (DHEA, 6 mg/100 g weight, Meilunbio, Dalian, China) for 20 days continuously. While the rats in control group were subcutaneously injected with equivoluminal phosphate buffer solution. The rats in EA + PCOS group were subjected to EA intervention (ST29 and SP6 acupuncture points, 2 Hz, 0.8–1.3 mA) every other day after the first injection with DHEA. EA was performed for 15 min each time in the first week, 20 min in the second and third week, and 25 min in the fourth and fifth week. All rats were killed after the 5-week EA intervention by injection with overdose of pentobarbital sodium. Animals were treated in accordance with Guide for the Care and Use of Laboratory Animals (8th edition, National Academies Press).

### Overexpression of SREBP1 in rats

The recombinant lentivirus containing SREBP1 gene (LV-SREBP1) and negative control lentivirus (LV-NC) were obtained from Wanleibio (Shenyang, China). The rats were intravenously injected with LV-SREBP1 or LV-NC (5 × 10^7^ TU per rat) once at the first day of DHEA injection.

### Inhibition of AMP-activated protein kinase (AMPK) signaling pathway in rats

To suppress the activation of AMPK signaling pathway in rats, the inhibitor of AMPK pathway compound C (10 mg/kg, MedChemExpress, USA) or equal volume vehicle was intraperitoneally injected into rats at 24 h before the euthanasia.

### HE staining

The ovarian tissues of rats after receiving the appropriate paraffin-embedding procedure, were cut into 5-μm sections using a slicer. Then the sections were subjected to dewaxing, rehydration, and routine HE staining. The images were observed at a magnification of 200 × under a light microscope (Olympus, Japan).

### ELISA

The serum levels of insulin, testosterone, luteinizing hormone (LH), and follicle-stimulating hormone (FSH) were assessed by commercialized ELISA kits (USCN Life Science, Wuhan, China). The contents of insulin, testosterone, LH, and FSH were calculated according to the standard curve.

### Immunofluorescence staining

Immunofluorescence staining was performed to detect the expression of SREBP1 in the ovarian tissues. The 5-μm sections of ovarian tissues were put into citrate antigen retrieval solution and heated in the microwave for 10 min. Subsequently, the sections were blocked in goat serum (Solarbio, Beijing, China) for 15 min. Primary SREBP1 antibody (1:100, Abcam, UK) was added to sections and incubated at 4 °C overnight. Then Cy3 labeled secondary antibody (1:200, Beyotime, Haimen, China) was applied at room temperature for 1 h. DAPI solution (Beyotime) was dropped to sections for nuclear counterstaining. The immunofluorescence staining of SREBP1 was observed under a fluorescence microscope (Olympus) at a magnification of 400 × .

### Primary culture of granulosa cells and treatment

The primary granulosa cells were isolated from rats as described previously [17]. Briefly, the female Sprague–Dawley and PCOS-like rats were accepted hypodermic injection with PMSG (15 IU per rat). Then the ovarian tissues were collected from rats under sterile conditions at 48 h after the injection. After washing with PBS for two times, the granulosa cells were released from the ovarian tissues by puncture using a 26-gauge needle. Then the collected granulosa cells were cultured in DMEM/F12 medium (Gibco, USA) containing 10% fetal bovine serum (Hyclone, USA) at 37 °C with 5% CO_2_.

To overexpress SREBP1, the granulosa cells were infected with LV-SREBP1 or LV-NC at multiplicity of infection (MOI) of 20. At 72 h after the infection, the granulosa cells were subjected to serum starvation for 12 h and then treated with 100 nM insulin for 5 min. Subsequently, the cells were obtained for further detection.

### Real-time PCR

RNApure Total RNA extraction kit (BioTeke, Beijing, China) was employed for total RNA isolation from the ovarian tissues. Then the total RNA was reverse transcribed using M-MLV reverse transcriptase (Takara Bio, Japan) to obtain cDNA. Real-time PCR was conducted using Taq HS Perfect Mix (Takara Bio) on a Real-Time Quantitative Thermal Block (BIONEER, Korea). The primer sequences are listed in Table 1.

**Table 1:** Oligonucleotide primer sets for real-time PCR

Name	Sequence (5′–3′)	Length
CYP17 F	TGGAGGTGATAAAGGGTT	18
CYP17 R	CGTCAGGCTGGAGATAGA	18
CYP19 F	GCCTGTCGTGGACTTGGT	18
CYP19 R	TAAATTCATTGGGCTTGG	18
SREBP1 F	CGACTACATCCGCTTCTTACA	21
SREBP1 R	AGGTTTCATGCCCTCCATA	19
β-actin F	CCACTGCCGCATCCTCTT	18
β-actin R	GGTCTTTACGGATGTCAACG	20

### Western blotting

RIPA lysis buffer (Beyotime) was adopted to acquire protein sample. Then the equal amount protein samples were loaded on 8–12% polyacrylamide gel for electrophoresis separation, followed by blotting onto a polyvinylidene difluoride membrane (Thermo Fisher Scientific, USA). Blocking was performed by incubation in 5% bovine serum albumin (BSA, Biosharp, Guangzhou, China) for 1 h. Then the membranes were probed with primary antibodies SREBP1 (1:1000, Proteintech, China), CYP17 (1:500, Proteintech), CYP19 (1:500, Proteintech), AMPKα (1:1000, Cell Signaling Technology, USA), p-AMPKα (1:1000, Cell Signaling Technology), p-AKT (1:2000, Cell Signaling Technology), AKT (1:1000, Cell Signaling Technology), p-IR (1:1000, Cell Signaling Technology), insulin receptor β (IR, 1:1000, Cell Signaling Technology), cytochrome C (1:1000, Abclonal, China), β-actin (1:2000, Proteintech) at 4 ℃ overnight. Goat anti-rabbit or anti-mouse IgG (1:10000, Proteintech) was used as secondary antibody. The antigen–antibody complexes were visualized with ECL Plus reagent (7 sea biotech, Shanghai, China).

### Detection of mitochondrial complex enzymes

The mitochondrial complex I, II, III, and IV activities in ovarian granulosa cells were determined with the commercial mitochondrial respiratory chain complex I, II, III, and IV assay kits (Solarbio) according to the manufacturer’s instructions.

### Mitochondrial membrane potential (MMP)

The MMP of ovarian granulosa cells was assessed with Mitochondrial Membrane Potential Assay Kit with JC-1 (Solarbio). Briefly, the mitochondria were isolated from ovarian granulosa cells by centrifugation (12,000 *g*, 10 min) at 4 °C twice. Then the isolated mitochondria were stained with JC-1 solution and measured using a fluorescence microplate reader (Tecan, Switzerland).

### Measurement of ATPase activity

The ATPase activity of ovarian granulosa cells was determined using ATP assay kit (Nanjing Jiancheng Bioengineering Institute, China) according to the standard procedure. The result was detected using a microplate reader (BioTek, USA) at 636 nm.

### Antioxidant-enzyme activity measurement

The CAT, SOD, and GPX activities of ovarian granulosa cells were assessed using Catalase (CAT) assay kit, Superoxide Dismutase (SOD) assay kit, and Glutathione Peroxidase (GSH-PX) assay kit following the operation manual, respectively. All the commercial kits were purchased from Nanjing Jiancheng Bioengineering Institute.

### Reactive oxygen species (ROS) level detection

The ROS level was determined using a ROS fluorescent probe-DHE (KeyGEN BioTECK, China). The isolated ovarian granulosa cells were washed with PBS, and probed with DHE buffer for 30 min at 37 °C. To remove the redundant DHE, the cells were washed with PBS for three times. The result was obtained on a flow cytometer (ACEA Biosciences, USA).

### Statistical analysis

All experimental data are expressed as the mean ± standard deviation. Differences among multiple groups were analyzed by one-way analysis of variance followed by Tukey’s multiple comparison test using GraphPad Prism 8 software. P value less than 0.05 was considered to have significant statistical difference.

## Results

### Effect of EA treatment on SREBP1 expression in PCOS-like rats

**Fig. 1:**
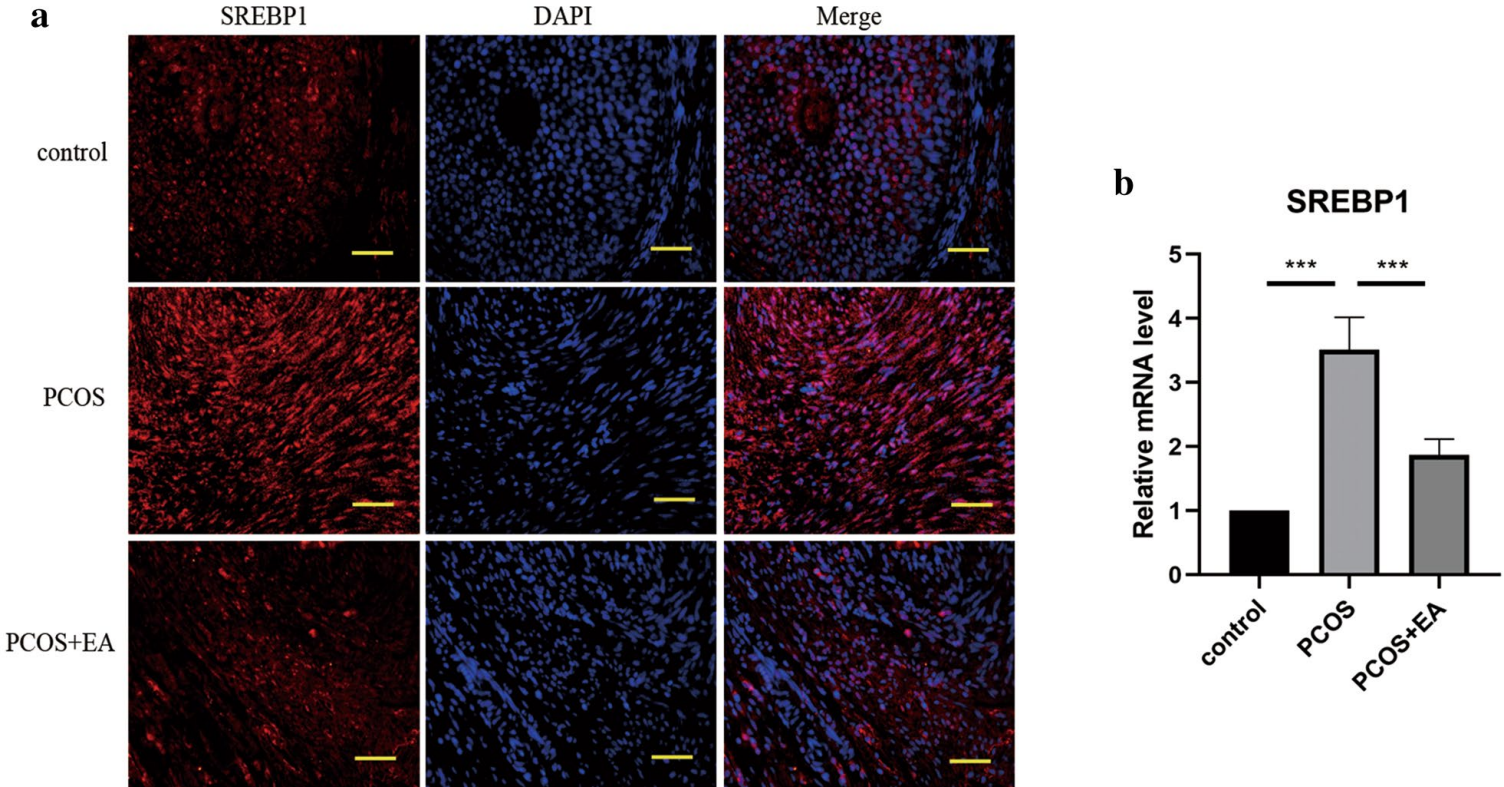
Effect of EA treatment on SREBP1 expression in PCOS-like rats. **a** The expression of SREBP1 in ovarian tissues was detected by immunofluorescent staining (magnification, 400 ×). Scar bar = 50 μm. **b** The mRNA level of SREBP1 in ovarian tissues was assessed by real-time PCR. All data are presented as mean ± standard deviation (n = 6). ***P < 0.001 versus the indicated group

First, the expression of SREBP1 in ovarian tissues of rats was observed by immunofluorescence staining. As presented in Fig. 1a, SREBP1 expression was obviously enhanced in the ovarian tissues of PCOS-like rats, as compared with control group. Whereas treatment with EA significantly suppressed DHEA-induced SREBP1 expression. Consistently, the mRNA level of SREBP1 in ovarian tissues was more than tripled in PCOS group, which could be remarkably repressed by EA treatment (Fig. 1b). Therefore, EA treatment restrained DHEA-induced the enhanced expression of SREBP1.

### EA intervention alleviated PCOS-like symptoms via regulating SREBP1

**Fig. 2:**
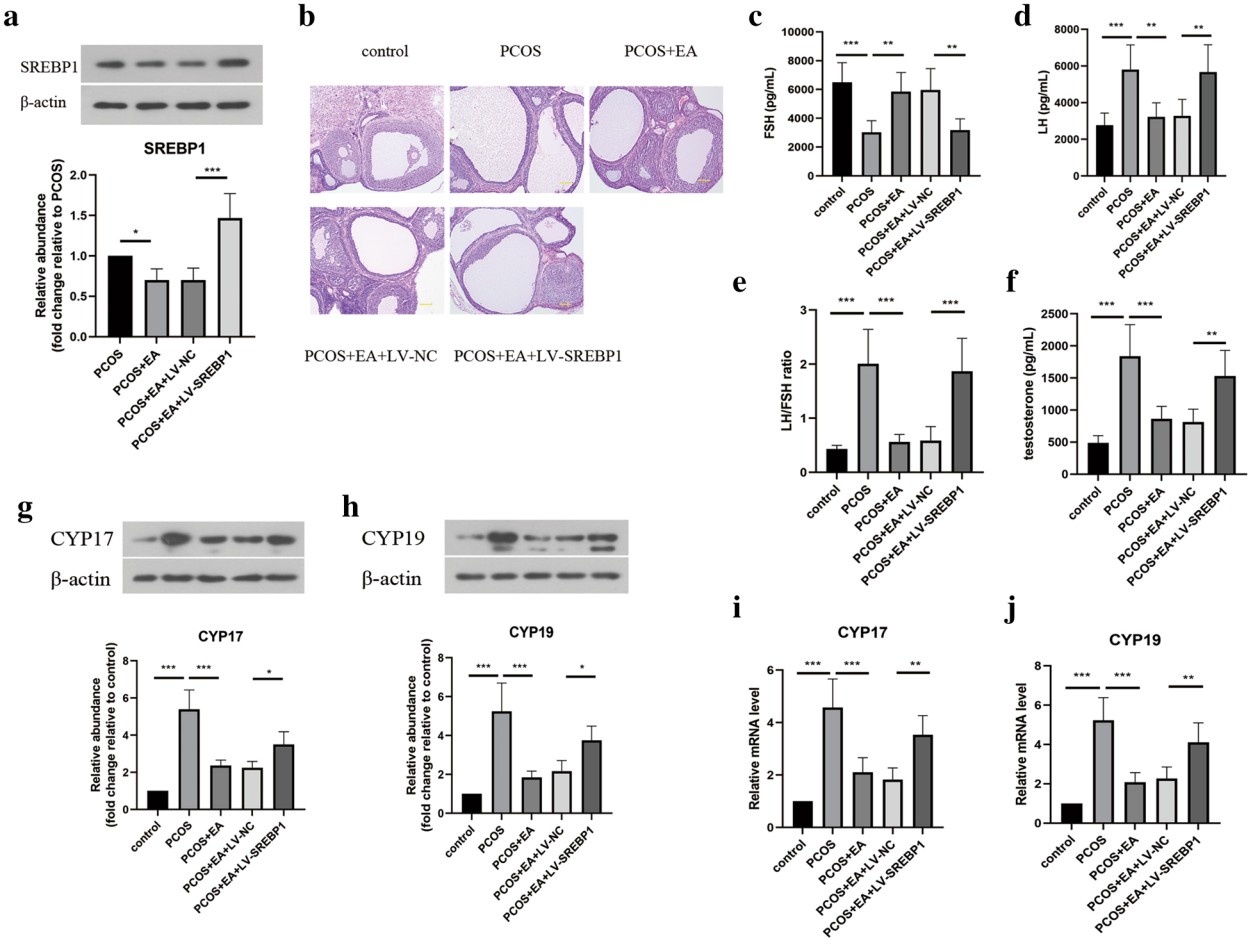
EA intervention alleviated PCOS-like symptoms via regulating SREBP1. **a** The protein level of SREBP1 in ovarian tissues from different groups was evaluated by Western blotting. **b** The pathological changes in ovarian tissues were observed by HE staining (magnification, 100 ×). Scar bar = 200 μm. The serum levels of FSH (**c**), LH (**d**), and testosterone (**f**) were determined by ELISA kits. **e** The ratio of LH/FSH was calculated and shown. The protein levels of CYP17 (**g**) and CYP19 (**h**) in ovarian tissues were detected by Western blotting. The mRNA expression of CYP17 (**i**) and CYP19 (**j**) in ovarian tissues was assessed by real-time PCR. All data are presented as mean ± standard deviation (n = 6). *P < 0.05, **P < 0.01, ***P < 0.001 versus the indicated group

Next, we determined whether SREBP1 participated in the beneficial effect of EA against PCOS-like symptoms in rats. To achieve this, lentivirus-mediated overexpression of SREBP1 in PCOS-like rats was performed. As shown in Fig. 2a, EA-induced the reduced protein level of SREBP1 in ovarian tissues was reversed by LV-SREBP1. In addition, the histopathologic changes of ovarian tissues were evaluated by HE staining. DHEA-induced multiple ovarian follicular cysts were relieved by EA intervention, however, overexpression of SREBP1 counteracted the therapeutic effect of EA (Fig. 2b). Moreover, SREBP1 reversed EA-induced increase in FSH level, and decreases in LH, testosterone levels and LH/FSH ratio (Fig. 2c–f). Furthermore, the protein and mRNA levels of CYP17 and CYP19 in ovarian tissues were significantly lowered by EA treatment, which were elevated by SREBP1 overexpression. These data indicated that overexpression of SREBP1 impaired the beneficial effect of EA on PCOS-like symptoms in rats.

### EA intervention restrained insulin resistance through regulating SREBP1 in PCOS-like rats

**Fig. 3:**
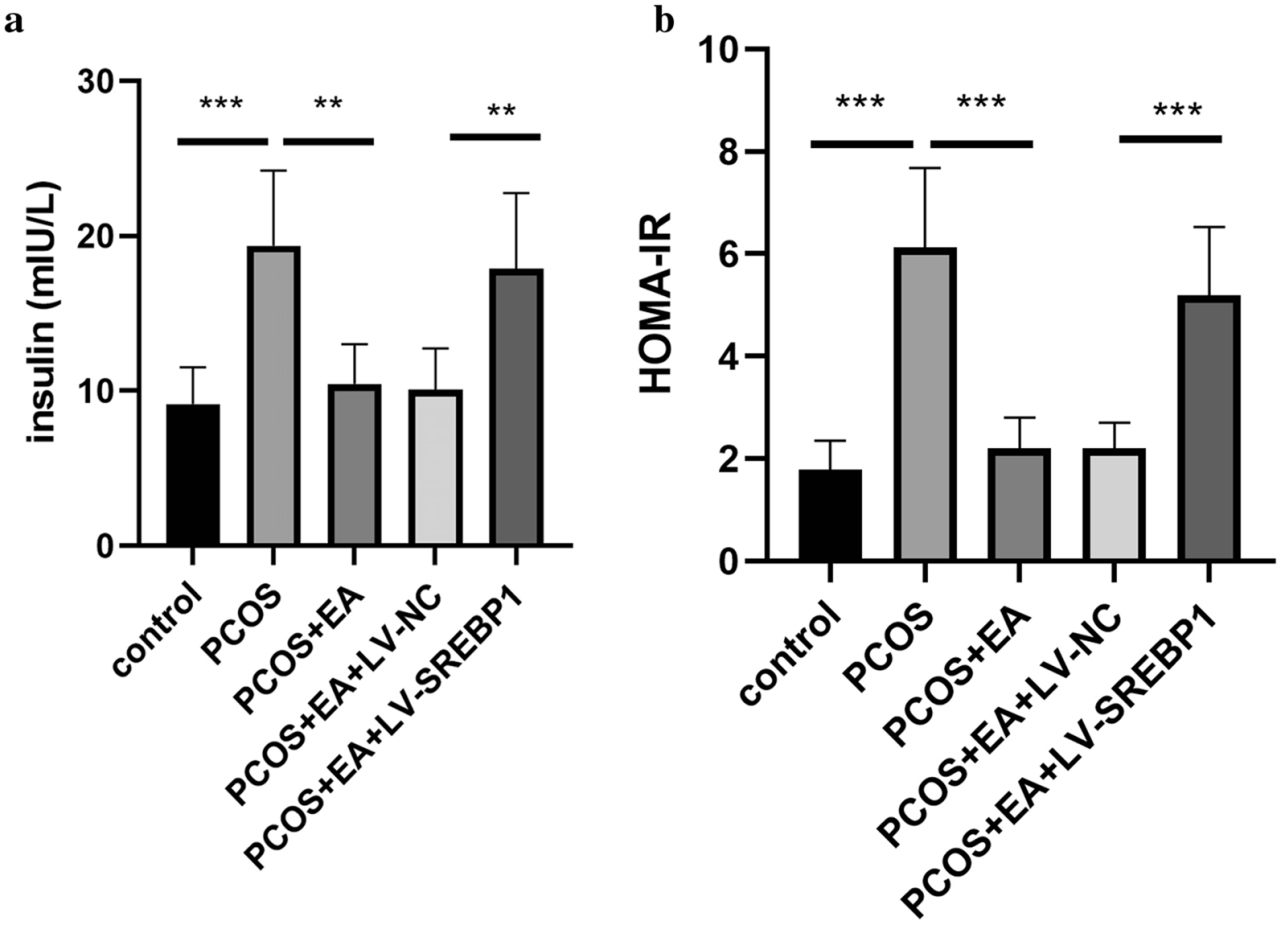
EA intervention restrained insulin resistance through regulating SREBP1 in PCOS-like rats. **a** The serum level of insulin was detected by ELISA. **b** The HOMA-IR index was calculated and shown. All data are presented as mean ± standard deviation (n = 6). **P < 0.01, ***P < 0.001 versus the indicated group

Since insulin resistance has been suggested as an important pathogenic mechanism of PCOS, we next evaluated whether SREBP1 was involved in EA-mediated regulation of insulin resistance in PCOS-like rats. As illustrated in Fig. 3a, the insulin level was strikingly reduced in EA treatment group as compared with PCOS group, whereas further overexpression of SREBP1 upregulated the insulin level. Additionally, EA intervention cut down the homeostatic model assessment of insulin resistance (HOMA-IR) index of PCOS-like rats, which was evidently reversed by SREBP1 overexpression. Thus, EA suppressed insulin resistance in PCOS-like rats via regulating SREBP1.

### EA regulated SREBP1 expression via AMPK pathway in PCOS-like rats

**Fig. 4:**
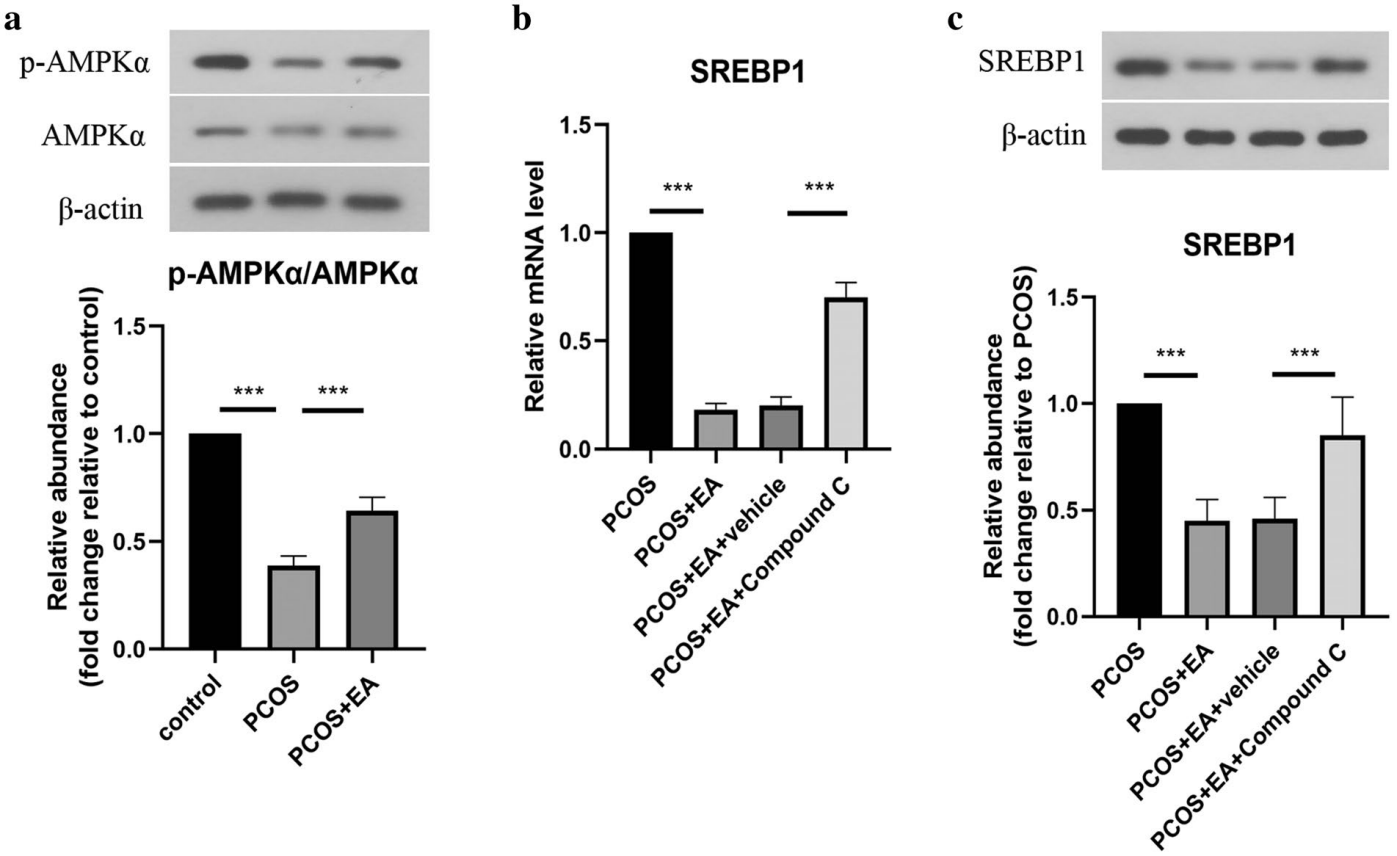
EA regulated SREBP1 expression via AMPK pathway in PCOS-like rats. **a** The protein levels of p-AMPKα and AMPKα in ovarian tissues were determined by Western blotting. **b** The mRNA expression of SREBP1 in ovarian tissues was assessed by real-time PCR. **c** The protein level of SREBP1 in ovarian tissues was evaluated by Western blotting. All data are presented as mean ± standard deviation (n = 6). ***P < 0.001 versus the indicated group

To further investigate the regulatory mechanism of EA on SREBP1 expression, we focused on AMPK signaling pathway. As shown in Fig. 4a, PCOS-mediated the inhibition of AMPK phosphorylation in ovarian tissues was remitted by EA treatment. Moreover, the decreased expression of SREBP1 in ovarian tissues induced by EA intervention was counteracted by compound C, an inhibitor of AMPK pathway. Therefore, EA repressed SREBP1 expression through activating AMPK signaling pathway in PCOS-like rats.

### Effect of SREBP1 on insulin pathway in ovarian granulosa cells

**Fig. 5:**
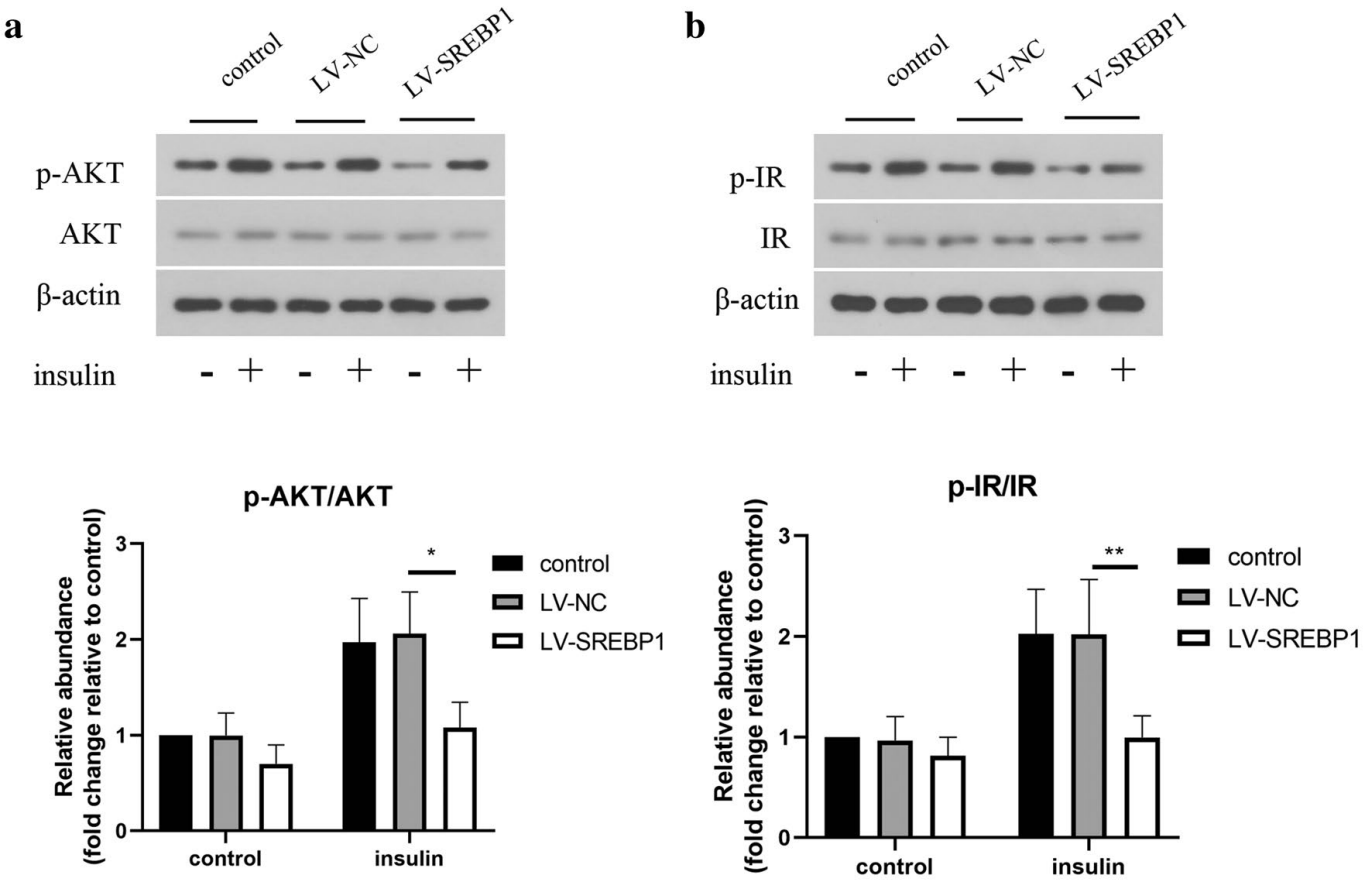
Effect of SREBP1 on insulin pathway in ovarian granulosa cells. **a**, **b** The protein levels of p-AKT, AKT, p-IR, and IR in primary ovarian granulosa cells isolated from rats were detected by Western blotting. All data are presented as mean ± standard deviation (n = 3). *P < 0.05, **P < 0.01 versus the indicated group

SREBP1 was overexpressed in ovarian granulosa cells by infection with LV-SREBP1. As presented in Fig. 5a and b, a remarkable increase in the phosphorylation of AKT and IR was observed after administration with insulin for 5 min. However, no significant changes were found in ERK and IR protein levels. Overexpression of SREBP1 inhibited AKT and IR phosphorylation induced by insulin.

### Effect of SREBP1 on mitochondrial dysfunction in ovarian granulosa cells

**Fig. 6:**
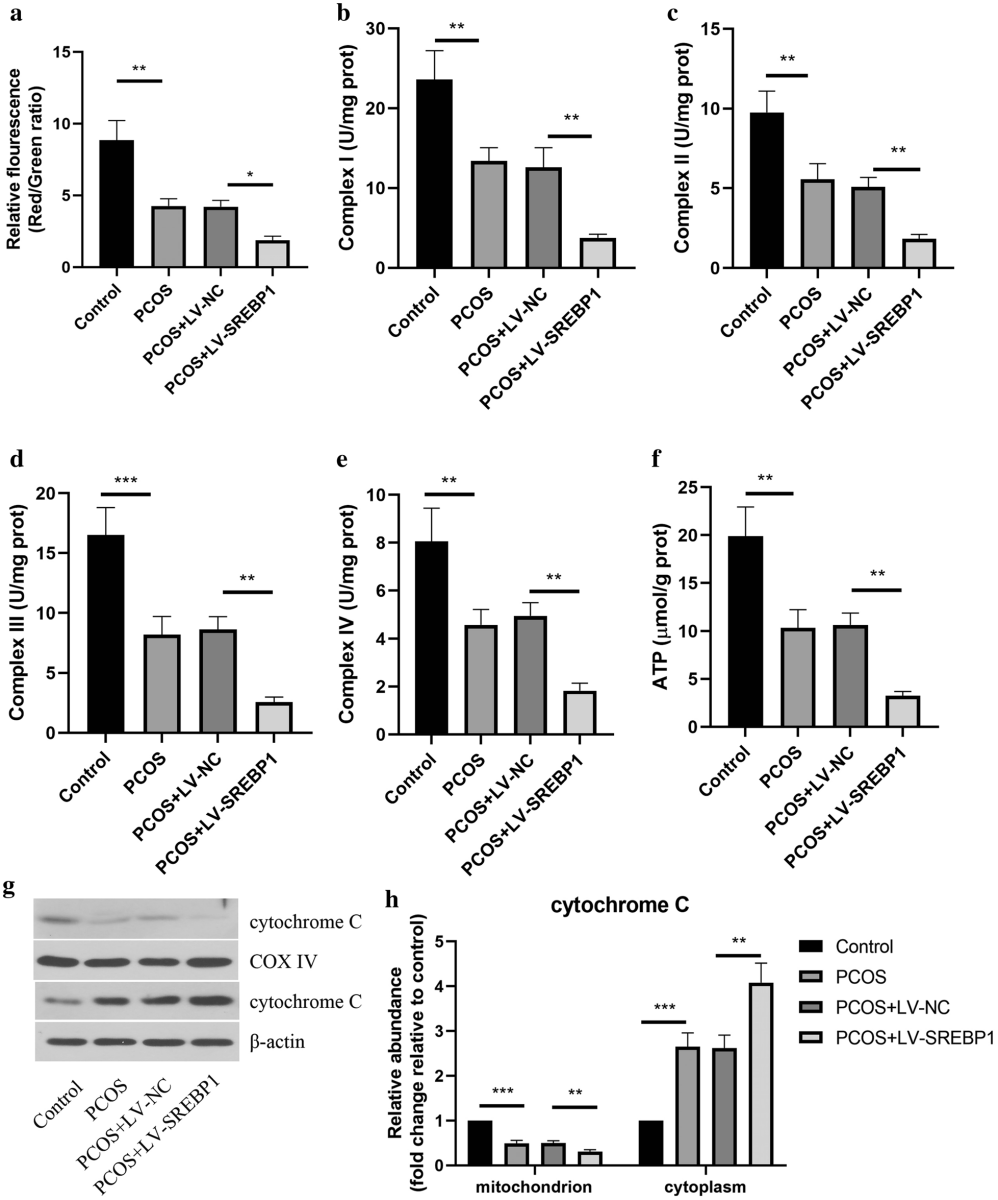
Effect of SREBP1 on mitochondrial dysfunction in ovarian granulosa cells. **a** JC-1 staining was adopted for detecting mitochondrial membrane potential. The activities of Complex I (**b**), Complex II (**c**), Complex III (**d**), Complex IV (**e**), and ATP (**f**) in ovarian granulosa cells were detected. **g**, **h** Western blotting for the protein level of cytochrome C in mitochondrion and cytoplasm of ovarian granulosa cells. All data are presented as mean ± standard deviation (n = 3). *P < 0.05, **P < 0.01, ***P < 0.001 versus the indicated group

The MMP of granulosa cells was assessed by JC-1 staining and shown in Fig. 6a. The PCOS-like granulosa cells exhibited evident decreased MMP, and overexpression of SREBP1 further enhanced this result. As shown in Fig. 6b–e, SREBP1 overexpression further reduced the decreased complex I, II, III, and IV activities of granulosa cells from PCOS-like ovaries. Furthermore, a significant decrease in ATP activity was observed in PCOS-like granulosa cells, and this change was strengthened by SREBP1 overexpression (Fig. 6f). In addition, the cytochrome C expression was decreased in the mitochondrion, but increased in the cytoplasm of PCOS-like granulosa cells, which was promoted by enforced expression of SREBP1 (Fig. 6g and h). These observations suggested that SREBP1 overexpression exacerbated mitochondrial dysfunction in PCOS-like granulosa cells.

### Effect of SREBP1 on oxidative stress in ovarian granulosa cells

**Fig. 7:**
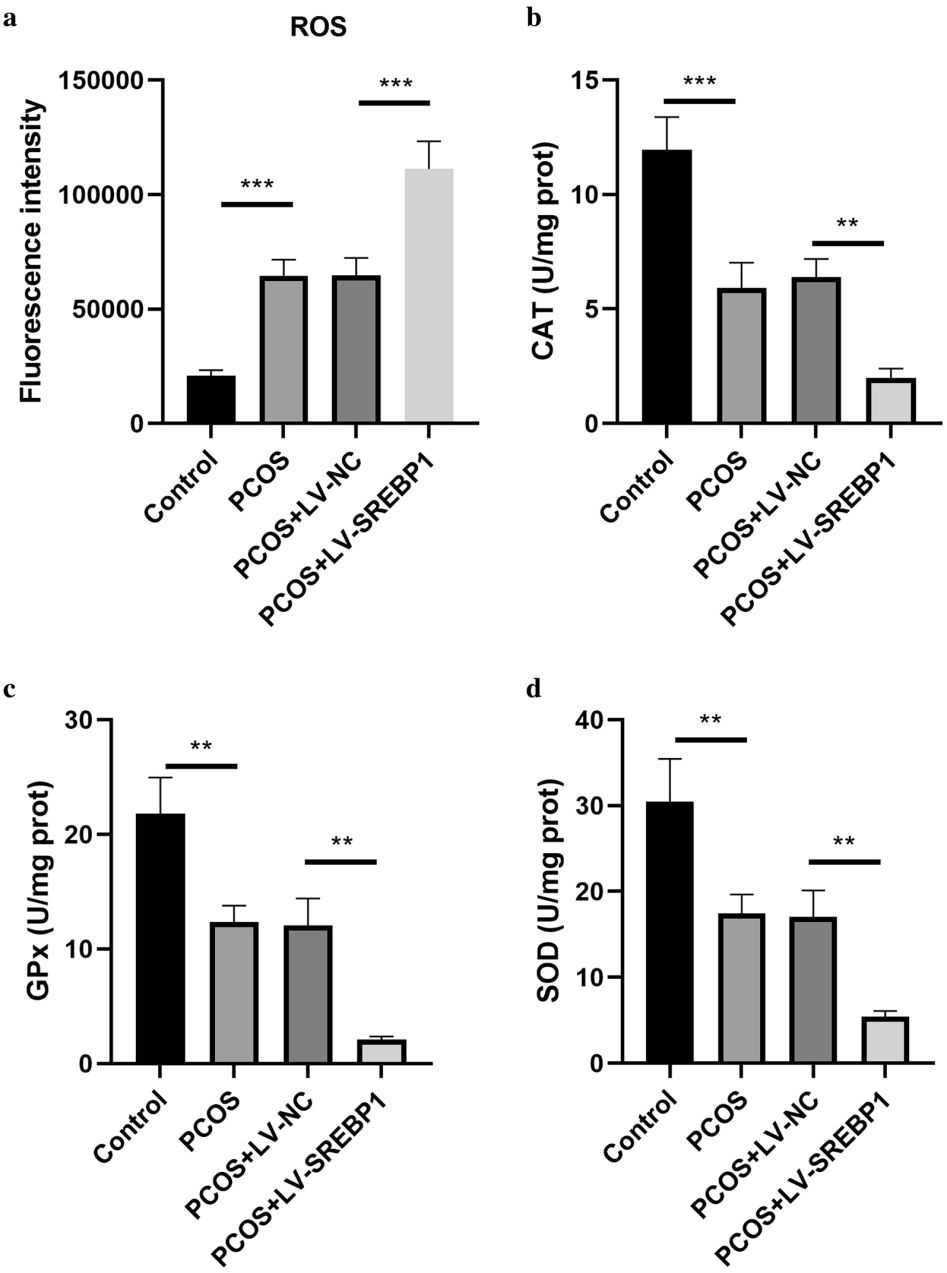
Effect of SREBP1 on oxidative stress in ovarian granulosa cells. The ROS level (**a**), and activities of CAT (**b**), GPX (**c**), and SOD (**d**) in ovarian granulosa cells were detected using the commercial kits. All data are presented as mean ± standard deviation (n = 3). **P < 0.01, ***P < 0.001 versus the indicated group

To assess the effect of SREBP1 on oxidative stress, the ROS level and activities of GPX, CAT and SOD were detected. As shown in Fig. 7a–d, the ROS level was up-regulated, and the CAT, GPX, and SOD activities were declined in PCOS-like granulosa cells. Overexpression of SREBP1 further reinforced the above changes. Thus, SREBP1 overexpression enhanced oxidative stress in PCOS-like granulosa cells.

## Discussion

Acupuncture, as an important therapy of traditional Chinese medicine, has been widely used and accepted because of no serious side-effect. For PCOS, acupuncture therapy has been demonstrated to have a confirmed therapeutic effectiveness. However, the precise mechanisms of acupuncture in PCOS are still not very clear. In this study, we mainly focused on the changes in the metabolic aspect of PCOS-like symptoms. Our results showed that EA treatment significantly restrained DHEA-induced SREBP1 expression. Overexpression of SREBP1 weakened the protective effect of EA on PCOS-like rats. Moreover, SREBP1 was involved in the regulation of EA in insulin resistance in PCOS-like rats. Mechanically, EA inhibited SREBP1 expression via activating AMPK signaling pathway in PCOS-like symptoms. SREBP1 overexpression suppressed insulin-stimulated activation of IR and AKT in ovarian granulosa cells. Therefore, our results further clarified the novel mechanisms of EA in PCOS-like symptoms.

It has been reported that the DHEA-PCOS animal model displays the features of human PCOS, including hyperandrogenism, aberrant maturation of ovarian follicles and anovulation [18–20]. Xi et al. confirmed that DHEA-challenged mice suffered whole-body and skeletal muscle insulin resistance, along with dysfunction of mitochondria [21]. In addition, reproductive and metabolic disturbances were found in DHEA-induced PCOS-like rats [22]. Therefore, the DHEA model was selected in this study to investigate EA-mediated protection against PCOS-like symptoms in rats. Many animal and human studies have provided some mechanistic evidence for the therapeutic effect of EA on PCOS. For instance, low-frequency EA reversed the epigenetic and transcriptional changes in the adipose tissues of PCOS patients [23]. Insulin sensitivity could be improved by electrical or manual acupuncture by regulating multiple metabolic Genes and signaling pathways in PCOS model [24]. EA could increase whole-body glucose uptake by activating nervous systems of PCOS women [25]. However, a recent study by Wu et al. found that EA treatment did not raise the live births among women with PCOS, suggesting that EA might led to metabolic changes rather that ovary changes [26]. In spite of these studies, the biological function and its detailed mechanisms of EA in PCOS need to be further investigated. SREBP1 is an important transcription factor for lipid metabolism regulation, which regulates the expression of many genes via binding to their promoter regions. A great deal of studies have demonstrated the aberrant expression of SREBP1 in multiple diseases, such as pancreatic cancer [27], diabetic nephropathy [28], non-alcoholic fatty liver disease [29] and so on. Recent evidence has also suggested that the increased expression of SREBP1 participates in the progression of PCOS [13, 30]. Consistent with this, our results showed that SREBP1 expression was raised in the ovarian tissues of PCOS-like rats. EA treatment inhibited SREBP1 expression in the ovarian tissues. Thus, inhibition of SREBP1 expression may take part in the therapeutic action of EA on PCOS.

To further evaluate whether EA treatment improved PCOS-like symptoms in rats via regulating SREBP1 expression, we overexpressed SREBP1 in rats by injecting LV-SREBP1. The disorder of sexual hormones has been confirmed to be closely related to the development of PCOS. The upregulated testosterone level indicates hyperandrogenism of PCOS patients [31]. Moreover, the elevated LH/FSH ratio is also a distinguishing hallmark of PCOS, which promotes the arrest of ovarian folliculogenesis [31, 32]. In the present study, EA intervention-mediated the inhibition in LH/FSH ratio and testosterone level was repressed by SREBP1 overexpression. Besides that, CYP17 and CYP19 are key enzymes in androgen biosynthesis, which were upregulated during the progression of PCOS [33, 34]. In this study, EA treatment suppressed CYP17 and CYP19 expression, which was reversed by SREBP1 overexpression. Therefore, EA treatment improved PCOS-like symptoms via suppressing SREBP1 expression.

Insulin resistance is another characteristic of PCOS, which frequently occurs in obese patients. It has been shown that insulin resistance promotes the production of male hormones, but lowers the level of sex hormone binding globulin, which leads to a dramatical increase in testosterone level in the body [35]. In addition, the increased LH/FSH ratio, testosterone and DHEA levels in PCOS women were caused by insulin resistance [36]. Thus, insulin resistance may cause hyperandrogenemia in PCOS. In the present study, EA treatment ameliorated insulin resistance as evidenced by decreasing serum insulin level and HOMA-IR index in PCOS-like rats, which was counteracted by SREBP1 overexpression. Previous studies also indicated that insulin action could be regulated in granulosa cells [37], and damaged insulin action may affect granulosa cells isolated from PCOS rats [38], indicating a close connection between insulin resistance and follicle formation. In normal condition, the released insulin binds to the insulin receptor and subsequently activates the phosphorylation of multiple downstream proteins, including IR and AKT [39]. Our data demonstrated that overexpression of SREBP1 restrained insulin-induced IR and AKT phosphorylation. These findings suggested that regulation of SREBP1 was involved in the protective mechanism of EA against insulin resistance in PCOS-like symptoms.

AMPK is recognized as an energy sensor that regulates fatty acid and cholesterol synthesis. Beyond that, AMPK also has been found to play pivotal roles in reproduction. A previous study has indicated that AMPK participates in the regulation of steroidogenesis in granulosa cells. Shabnam et al. suggested that activation of AMPK regulated steroidogenesis in granulosa cells from PCOS rats [40]. Moreover, growing evidence demonstrated that SREBP1, as a downstream molecule, regulated by AMPK [41–43]. In this study, we found that EA intervention facilitated the activation of AMPK pathway in PCOS-like rats. EA-induced inhibition of SREBP1 expression was repressed by AMPK inhibitor. Thus, EA treatment inhibited SREBP1 expression through activating AMPK pathway in PCOS-like symptoms.

Increasing evidence has indicated that mitochondrial dysfunction participates in the pathogenesis of PCOS. It has been confirmed that mitochondrial dysfunction is closed related with the pathological features of PCOS, including insulin resistance and hyperandrogenism [44]. Since mitochondrial function is critical for energy supply, the disorder of mitochondria may lead to reduced production of ATP [45]. Moreover, mitochondrial dysfunction results in excessive ROS production, which causes oxidative stress, another important pathogenesis of PCOS [46]. Typically, the activities of antioxidant enzymes such as SOD, CAT and GPX have been verified to be decreased in PCOS model [47]. Consistent with these observations, in this study the mitochondrial dysfunction and oxidative stress were found in PCOS-like granulosa cells, which were further aggravated by SREBP1 overexpression. Therefore, SREBP1 participated in mitochondrial dysfunction and oxidative stress during the progression of PCOS.

We realized that there are some limitations in our study. The changes in downstream receptors may be attributed to metabolic or neural origin cardiovascular disturbances of PCOS, which have not been elucidated in this study. In the future study, we will focus on these issues, which can more comprehensively understand the mechanisms of EA.

## Conclusions

This research suggests that EA intervention attenuates hyperandrogenemia, insulin resistance, mitochondrial dysfunction and oxidative stress in PCOS-like model via inhibiting the expression of lipid metabolism regulator SREBP1. AMPK pathway is activated by EA, which participates in the regulation of SREBP1. Our results shed new light on the protective mechanisms of EA for the PCOS treatment.

## Data Availability

The datasets used or analyzed during the current study are available from the corresponding author on reasonable request.

## Abbreviations

PCOS: Polycystic ovary syndrome
EA: Electroacupuncture
DHEA: Dehydroepiandrosterone
SREBP1: Sterol regulatory element binding protein-1
IR: Insulin receptor β
AMPK: AMP-activated protein kinase
LH: Luteinizing hormone
FSH: Follicle-stimulating hormone
MOI: Multiplicity of infection
